# REGISTRI: Regorafenib in first-line of *KIT/PDGFRA* wild type metastatic GIST: a collaborative Spanish (GEIS), Italian (ISG) and French Sarcoma Group (FSG) phase II trial

**DOI:** 10.1186/s12943-023-01832-9

**Published:** 2023-08-09

**Authors:** Javier Martin-Broto, Claudia Valverde, Nadia Hindi, Bruno Vincenzi, Javier Martinez-Trufero, Giovanni Grignani, Antoine Italiano, Javier Lavernia, Ana Vallejo, Paolo Dei Tos, Francois Le Loarer, Ricardo Gonzalez-Campora, Rafael Ramos, Diana Hernández-Jover, Antonio Gutierrez, Cesar Serrano, Maria Monteagudo, Rocio Letón, Mercedes Robledo, David S. Moura, Marta Martin-Ruiz, Jose A. López-Guerrero, Julia Cruz, Antonio Fernandez-Serra, Jean-Yves Blay, Elena Fumagalli, Virginia Martinez-Marin

**Affiliations:** 1 Health Research Institute-Fundación Jiménez Díaz University Hospital, Universidad Autonoma de Madrid (IIS-FJD, UAM), 28040 Madrid, Spain; 2 Medical Oncology Department, Fundación Jimenez Diaz University Hospital, Av. de los Reyes Católicos, 2, 28040 Madrid, Spain; 3 General de Villalba University Hospital, 28400 Madrid, Spain; 4 Medical Oncology department, Vall d’Hebron University Hospital, 08035 Barcelona, Spain; 5 Medical Oncology, University Campus Bio-Medico and Fondazione Policlinico Universitario Campus Bio-Medico, 00128 Rome, Italy; 6 Medical Oncology Department, University Hospital Miguel Servet, 50009 Zaragoza, Spain; 7 Medical Oncology Unit, Città della Salute e della Scienza Hospital, 10126 Turin, Italy; 8 Medical Oncology department, Institute Bergonié, 33076 Bordeaux, France; 9 Medical Oncology department, Fundación Instituto Valenciano de Oncologia, 46009 Valencia, Spain; 10 Pathology department, Hospital Regional Universitario de Malaga, 29010 Malaga, Spain; 11 Department of Medicine, School of Medicine, University of Padua, 35122 Padua, Italy; 12 Pathology department, Institute Bergonié, 33076 Bordeaux, France; 13 Pathology department, Hospital Quironsalud, 14004 Cordoba, Cordoba, Spain; 14 Pathology department, University Hospital Son Espases, 07120 Mallorca, Spain; 15 Radiology department, Sant Pau University Hospital, 08025 Barcelona, Spain; 16 Hematology department, University Hospital Son Espases, 07120 Mallorca, Spain; 17 Hereditary Endocrine Cancer Group, Human Cancer Genetics Program, Spanish National Cancer Research Centre (CNIO), Madrid, Spain; 18 Centro de Investigación Biomédica en Red de Enfermedades Raras (CIBERER), Institute of Health Carlos III (ISCIII), Madrid, Spain; 19 Molecular Biology department, Fundación Instituto Valenciano de Oncologia, 46009 Valencia, Spain; 20 Pathology department, Fundación Instituto Valenciano de Oncologia, 46009 Valencia, Spain; 21 Medicine Department, Centre Léon Bérard, 69008 Lyon, France; 22 Medicine Department, Fondazione IRCCS - Istituto Nazionale dei Tumori, 20133 Milan, Italy; 23 Department of Medical Oncology, Hospital Universitario La Paz-IdiPAZ, P. Castellana, 261, 28046 Madrid, Spain

**Keywords:** Wild type GIST, SDH: Regorafenib, Biomarker, Clinical trial

## Abstract

**Background:**

Approximately 15% of adult GIST patients harbor tumors that are wild-type for KIT and PDGFRα genes (KP-wtGIST). These tumors usually have SDH deficiencies, exhibit a more indolent behavior and are resistant to imatinib. Underlying oncogenic mechanisms in KP-wtGIST include overexpression of HIF1α high IGFR signaling through the MAPK pathway or BRAF activating mutation, among others. As regorafenib inhibits these signaling pathways, it was hypothesized that it could be more active as upfront therapy in advanced KP-wtGIST.

**Methods:**

Adult patients with advanced KP-wtGIST after central confirmation by NGS, naïve of systemic treatment for advanced disease, were included in this international phase II trial. Eligible patients received regorafenib 160 mg per day for 21 days every 28 days. The primary endpoint was disease control rate (DCR), according to RECIST 1.1 at 12 weeks by central radiological assessment.

**Results:**

From May 2016 to October 2020, 30 patients were identified as KP-wtGIST by Sanger sequencing and 16 were confirmed by central molecular screening with NGS. Finally, 15 were enrolled and received regorafenib. The study was prematurely closed due to the low accrual worsened by COVID outbreak. The DCR at 12 weeks was 86.7% by central assessment. A subset of 60% experienced some tumor shrinkage, with partial responses and stabilization observed in 13% and 87% respectively, by central assessment. SDH-deficient GIST showed better clinical outcome than other KP-wtGIST.

**Conclusions:**

Regorafenib activity in KP-wtGIST compares favorably with other tyrosine kinase inhibitors, especially in the SDH-deficient GIST subset and it should be taken into consideration as upfront therapy of advanced KP-wtGIST.

**Trial registration:**

ClinicalTrials.gov Identifier: NCT02638766.

**Supplementary Information:**

The online version contains supplementary material available at 10.1186/s12943-023-01832-9.

## Introduction

Gastrointestinal stromal tumors (GIST) lacking mutations in *KIT* or *PDGFRα* genes, formerly known as wild-type GIST, represent around 10–15% of GIST in adults, while they represent 85% in pediatric population. This subset of GIST, henceforth referred to as *KIT*/*PDGFRA* wild-type (KP-wGIST), was considered to have low to null responsiveness to imatinib [1]. In the pre-era of next generation sequencing (NGS), an overall response rate of 25% and 37% was reported for KP-wGIST in patients enrolled in two different randomized phase III trials [2]. Furthermore, significantly shorter progression-free survival and overall survival was found in KP-wGIST, with hazard ratios of 0.37 and 0.40 respectively, when compared with exon 11-mutant cases [3].

Although KP-wGIST constitute a heterogeneous group, they do display more chromosome stability than mutated GIST, hardly exhibiting genomic imbalances at all. A large recent series of 95 KP-wGIST cases found that succinate dehydrogenase (SDH)-competent GIST represented 12%, whereas SDH-deficient GIST was 88%. Within the latter, detectable by negative SDHB immunohistochemistry, 75% had *SDH* mutations and 25% had methylation of the SDHC promoter [4]. The SDH-competent subset encompasses a wide spectrum of gene mutations including more frequently *BRAF*, *NF1*, and less frequently *CBL*, *ARID1A* or *NTRK* genes, the later as fusion-type genomic alteration especially *ETV6-NTRK3* [1].

Neo-angiogenesis activation is a recognized underlying signaling in the most frequent KP-wGIST, SDH-deficient GISTs. Loss of function of SDH owing to mutational inactivation, or promoter inactivation by methylation, leads to the cytoplasmic accumulation of succinate which downregulates prolyl hydroxylase. This enzyme has a negative regulator role as regards HIF1α since it promotes its proteasomal degradation. Increased levels of HIF1α can enter the nucleus and activate the transcription of vascular endothelial growth factors (VEGFR). In fact, VEGFR expression is higher in KP-wGIST than in *KIT* mutant GISTs [5]. Additionally, IGFR1 is upregulated in the context of SDH-deficient GIST by mechanisms not yet fully understood. IGFR signals through both the MAPK and PI3K-AKT pathways. Similarly, *BRAF* and *NF1*-mutant GISTs signal downstream through MAPK.

Regorafenib is an oral multikinase inhibitor of angiogenic (VEGFR1-3, TIE2), stromal (PDGFR-b, FGFR), and oncogenic kinases (KIT, RET, RAF-1, BRAF, and BRAFV600E). A phase III trial showed the superiority of regorafenib over placebo in advanced GIST patients progressing to imatinib and sunitinib, with a median progression-free survival of 4.8 months vs. 0.9 months [6]. This study led to the registration of regorafenib in third-line treatment of advanced GIST patients.

Regorafenib is a potent inhibitor of KIT and downstream phosphorylation (AKT, MAPK, S6). Furthermore, early interstitial Cajal cell (ICC) progenitors have a phenotype of KIT^low^CD44+CD34 + IGFR + while committed lineage progenitors have KIT^high^CD44+CD34-IGFR-. Unlike mature or more committed lineage ICCs, the KIT^low^CD44+CD34 + IGFR + display resistance to Imatinib in spite of Kit signaling pathway activation. Interestingly, at least 50% of KP-wGIST overexpress IGFR1. This overexpression could correlate with SDH deficiency due to the IGF autocrine loop [7]. IGFR signals through the MAPK pathway, among other signaling pathways, that might be inhibited by regorafenib at different levels. Taken together, the previous information indicates that regorafenib may be more advantageous than imatinib for advanced KP-wGIST patients as upfront therapy.

A phase II trial was then designed to explore regorafenib in the first-line treatment of advanced KP-wGIST, screened by NGS, with the cooperation of Spanish, French and Italian sarcoma groups.

## Methods

### Study design and participants

In this phase II trial, adult patients (≥ 18 years) with metastatic KP-wGIST who were naïve of systemic treatment for advanced disease, were enrolled in 15 tertiary, expert sarcoma centers in Spain, France and Italy. Other relevant inclusion criteria were histological confirmation by central pathology review, as well as central molecular confirmation of wild-type condition in *KIT* and *PDGFRA* genes by next generation sequencing technology. Subjects, with an Eastern Cooperative Oncology Group (ECOG) Performance Status of 0 or 1, must have at least one measurable lesion according to RECIST v1.1 criteria. Patients had to provide written, informed consent before study-specific procedures or assessments were made and had to be willing to comply with treatment and follow-up. Informed consent was obtained before the start of the screening process. Approval from the ethics committee of each participating center was obtained before study initiation.

Some relevant exclusion criteria were any prior systemic treatment for metastatic GIST. Patients that have received imatinib in an adjuvant setting are eligible only if they have relapsed after a minimum of 2 years from ending treatment with imatinib. Cancers other than GIST within 5 years prior to randomization except for curatively treated cervical cancer in situ, non-melanoma skin cancer, and non-invasive superficial bladder tumors. A protocol amendment allowed for the accrual of patients previously treated with imatinib.

Patients received 160 mg (4 × 40 mg film-coated tablets) of regorafenib, once a day for 3 weeks, in 4-week cycles. If dose reduction of regorafenib was necessary because of toxicity, the dose would be reduced stepwise by 40 mg at each step. Two dose-level reductions were considered, 120 mg (-1) and 80 mg (-2) per day. If the subject needed more than 2 dose-level reductions, treatment should be discontinued. If the toxicity was abated with dose reduction up to grade 0 or 1 and, at the discretion of the investigator, was considered safe, the regorafenib dose would then be increased stepwise. Dose interruptions were planned and detailed by the protocol for general toxicities, and specifically for skin, blood pressure and liver toxicities.

Treatment with regorafenib was continued until any of the following events occurred: RECIST progression, unacceptable toxicity, patient considered non-compliant with the protocol requirements by the investigator or sponsor, withdrawal of consent, or a delay in regorafenib administration of longer than 4 weeks. Adverse effects were graded according to CTCAE 4.03 and were monitored once every 4 weeks. Radiological assessments of target and non-target lesions were evaluated every 8 weeks by CT scans. Tumor density was determined by indicating the CT attenuation coefficient in Hounsfield units (HU) captured in portal phase. Central radiology review was mandatory at the end of the study. All centers had to upload their CT scans, in an anonymous way, to a web-based imaging platform.

The main endpoint was disease control rate (DCR) lasting for at least 12 weeks, and secondary endpoints were progression-free survival (PFS), overall survival (OS), overall response rate (ORR) by RECIST 1.1 and Choi criteria, safety and quality of life.

### Statistical design

To estimate the sample size, a single-stage phase II clinical trial based on exact (binomial) tests has been used. The main endpoint will be to assess DCR taking into account the historical control with imatinib in wild-type GIST. Therefore, in H0, p0 will be taken as 73% (0% CR + 23% PR + 50% SD) [8]. H1 would be to achieve a DCR of p1 = 90% with regorafenib. Using error rates alpha 0.1 and beta 0.20. The total number of patients is estimated at 23 evaluable patients.

### Central pathology review and DNA sequencing

An external central pathology review was performed based on tumor hematoxylin-eosin staining and positive immunostaining for DOG1 and/or CD117 (c-KIT) antibodies (Roche; Basel, Switzerland).

For *KIT* and *PDGFRA* mutational screening, DNA was isolated from 3 sections of 5-µm thick FFPE samples using the QIAamp® DNA Investigator kit (Qiagen, Hilden, Germany) as indicated in the manufacturers’ instructions. When the tumor content was lower than 30%, a microdissection of the area with higher tumor content was performed. DNA concentration was fluorometrically measured by using the Quant-iT™ PicoGreen™ dsDNA Assay Kit (ThermoFisher, Hamburg, Germany). The libraries were prepared using the Solid Tumor Solution (STS) gene panel from Sophia GeneticsTM, following the manufacturer’s recommendations. Briefly, 50 ng of extracted DNA were enzymatically fragmented to a size between 200 and 800 bp. The custom panel interrogates hot spots from 42 actionable genes associated with solid tumours, including exons 8–11, 13, 17 and 18 of *KIT* (NM_000222.2) and exons 12, 14 and 18 of *PDGFRA* (NM_006206.5). Pooled libraries were sequenced (2 × 150 bp, paired-end) on a NextSeq550 instrument (Illumina™). Variant calling and annotation were performed by Sophia DDMTM platform. Variants were selected based on the following parameters: coverage > 600x, allelic frequency (AF) > 10% and annotation of pathogenic and/or likely pathogenic. Visualization of variants was performed with the Integrative genomic viewer (IGV) software from de Broad Institute (https://software.broadinstitute.org/software/igv/).

For genetic testing beyond *KIT* and *PDGFRA*, *post hoc* somatic mutational status characterization was performed using a customized NGS panel. Briefly, the targeted gene panel was designed using an AmpliSeq Custom DNA Panel (Illumina, San Diego, CA, USA), including the following genes: *VHL, RET, SDHA, SDHB, SDHC, SDHD, SDHAF2, SDHAF1, MAX, HIF1A* (exon 12), *HIF2A* (exon 12), *TMEM127, HRAS, KRAS, NF1, GOT2, FH, MDH2, SLC25A11, DNMT3A* (exon 8), *DLST* (exon 14), *MERTK* (exon 17), *IDH1, IDH2, CSDE1, EGLN1, EGLN2, BRAF* (exon 15), *MET* (exons 14–21), *FGFR1* (exons 12 and 14), *KIF1B, CDKN1B, MEN1, PTEN, H3F3a* and *ATRX*. The panel was used according to the manufacturer’s instructions, starting with 200ng of DNA. Interpretation of variants was performed following the recommendations of the NGS in PPGL Study Group and the American College of Medical Genetics and Genomics-Association for Molecular Pathology guidelines, and mutations detected were confirmed by Sanger sequencing.

### Statistical design for translational study

Variables following binomial distributions (i.e.: response rate), were expressed as frequencies and percentages. Comparisons between qualitative variables were made using the Fisher Exact Test or Chi-square, with Yates’ continuity correction when necessary. Comparisons between quantitative and qualitative variables were performed through non-parametric tests (U of Mann-Whitney or Kruskal-Wallis). Time to event variables (OS and PFS) were measured from the date of therapy onset and were estimated according to the Kaplan-Meier method. Comparisons between the variables of interest were performed by log-rank test. Median follow-up was calculated using the reverse Kaplan-Meier estimator. All p-values reported were 2-sided, and statistical significance was defined at p < 0.05.

## Results

From May 2016 to October 2020, 30 advanced GIST patients, with no mutations found by Sanger sequencing in *KIT* or *PDGFRA* genes, were centrally screened using NGS. Sixteen patients were confirmed by NGS as KP-wGIST and were eligible for the study, although one patient declined to participate as the diagnosis was coincident with the SARS-CoV-2 pandemic. Thus, a total of 15 patients were enrolled and received regorafenib, less than initially estimated since the trial was prematurely closed due to the low accrual worsened by the COVID outbreak (Additional File 1). The clinical cutoff for the final data analyses was November 9, 2021. At that time 2 patients were still receiving regorafenib (25 + and 43 + months from treatment initiation), whereas 13 had discontinued the treatment, 8 because of progression, 2 patients refused to continue with regorafenib, 2 patients due to toxicity (one with G4 Steven-Johnson syndrome at day 13 of the first cycle and another with G5 gastric hemorrhage at day 8 of the first cycle) and 1 patient because of surgical rescue. A 16-year old female patient was allowed to be accrued, through a waiver, after obtaining her signed informed consent along with the agreement of her parents.

The median age was 57 years (16–72), without any predominant gender (8 female and 7 male patients). Tumor locations were distributed as follows: gastric 10 (67%), intestinal 2 (13%) and mesenteric 3 (20%). In 9 patients, an SDH-deficient GIST was detected by immunohistochemistry, 5 of them harbored mutations in the *SDH* gene and 1 had a variant of uncertain significance (VUS), according to ClinVar, in the fumarate hydratase (*FH)* gene (Additional File 2). In 6 patients with SDH-proficient GIST, mutations were found in *BRAF*, *NF1* and *MAX* genes in one patient each. No mutations were detected in 2 patients and 1 case was not evaluable due to an exhausted paraffin block. All 9 SDH-deficient GISTs had a gastric location. At baseline 13 patients were metastatic and 2 were locally-advanced, considered unresectable. Other demographics are depicted in Table 1.

**Table 1:** Patients demographics (n = 15)

	N (%)
Median age at diagnosis (range)	56 (16–72)
Median age at enrollment (range)	57 (16–72)
Gender	
- Male - Female	7 (47%) 8 (53%)
ECOG at baseline:	
- 0 - 1	10 (67%) 5 (33%)
Median tumor size (range) at diagnosis (mm)	60 (13–250)
Tumor extension at diagnosis:	
- Localized - Locally advanced - Metastatic	6 (40%) 2 (13%) 7 (47%)
Tumor extension at enrollment:	
- Locally advanced - Metastatic	2 (13%) 13 (87%)
IHC-SDH:	
- Positive - Negative - Not available	5 (33%) 8 (53%) 2 (13%)
Any mutation:	
- Yes - No - Not available	10 (67%) 4 (27%) 1 (6%)
SHD mutation:	
- SDH - Other gene - No mutation - Not available	6 (40%) 4 (27%) 4 (27%) 1 (6%)
Any alteration in any gene:	
- Yes - No	12 (80%) 3 (20%)
Any alteration in SDH:	
- Yes - No	9 (60%) 6 (40%)

A total of 220 1-month cycles of treatment were given to the 15 enrolled patients with a median of 9 (0.3–55) cycles per patient. Dose reductions and dose interruptions were registered in 8 (53%) and 14 (93%) patients, respectively. The median dose intensity for regorafenib was 74% (4-100%). At baseline, 5 (33%) patients had previously received imatinib, 4 of which were in the adjuvant setting. The patient that had received imatinib in the advanced disease was accrued as RECIST progression, as were the 6 cases that had a metastatic recurrence. The remaining 8 cases that presented as advanced disease were enrolled at the time of diagnosis without a watch-and-wait period.

The most frequent secondary adverse events of any grade in the 15 accrued patients were hypertension (66.7%), fatigue (60%), diarrhea (46.7%), anorexia (40%) and cutaneous rash (40%). The most common grade 3 and 4 toxicities were increased alanine aminotransferase concentration (20%), increased aspartate aminotransferase (13.3%), hypertension (13.3%), palmar-plantar erythrodysesthesia (13.3%) and anorexia (13.3%). While the most frequent hematological toxicity was anemia (20%), followed by lymphocytopenia (13.3%). No grade 3 or 4 neutropenia or thrombocytopenia were observed (Additional File 3).

**Fig. 1:**
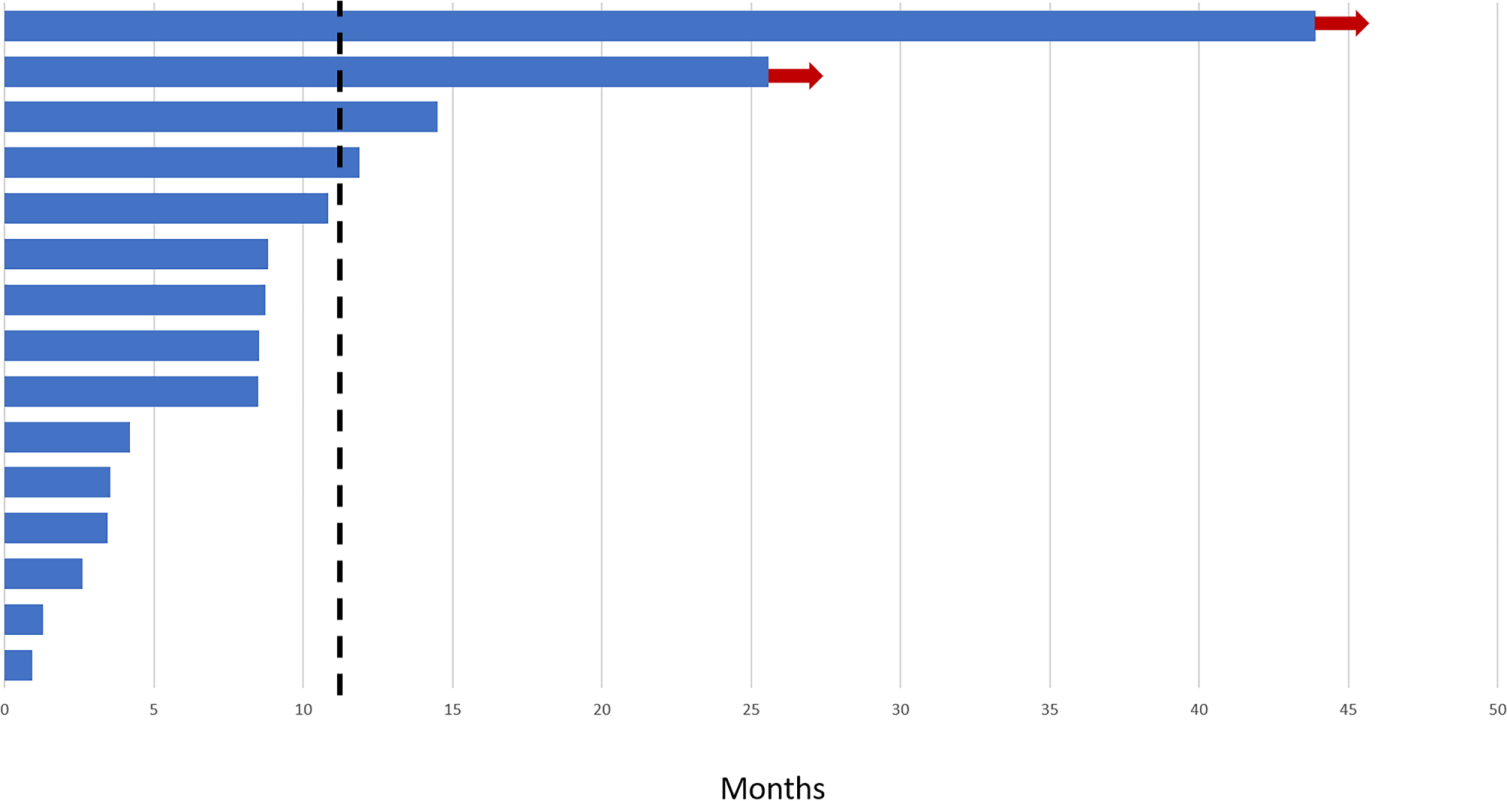
Swimmers plot for progression-free survival according to RECIST central radiological review. Each bar represents one patient. Red arrows identify patients with absence of progression at the last radiological follow up. The vertical line represents the median progression-free survival for the whole series

Based on RECIST criteria and central radiology assessment, 2 (13%) patients had partial response (Additional File 5), and 13 (87%) had stable disease. DCR lasting at least 12 weeks, by central assessment, was 86.7% (Fig. 1). Nine (60%) patients achieved some dimensional decrease following regorafenib, being the median and mean of tumor shrinkage in these patients of 12 and 16.5% respectively (Additional Fig. 6). Furthermore, following Choi criteria, 8 (57%) patients had partial response, and 6 (43%) had stable disease out of 14 evaluable patients for this criterion (Additional File 6). The median of tumor density decrease was 20%.

With a median follow up of 42 months (95% CI 28–56), the median of PFS in accordance with central review was 11 months (95% CI, 0.79–21.21) by RECIST and 14.9 months (95% CI, 0–47) by Choi criteria (Additional File 7). The median of OS was not reached at the time of analysis.

No significant correlation was found between SDH-deficient GIST and clinical outcome with the exception of a longer PFS, according to Choi criteria (Additional File 8 and Additional File 9) that favored the SDH-deficient subset, 11 months (95% CI, 3.3–18.6) vs. not reached (NR)(p = 0.04). However, SDH-deficient GIST also displayed a trend towards a longer PFS following RECIST central assessment NR vs. 11 months (p = 0.20). In addition, SDH-deficient GIST showed a trend toward a longer OS compared with SDH-proficient GIST, NR vs. 28.7 months (p = 0.31) (Additional File 8). All SDH-deficient GIST showed KIT immunoreactivity, while 8 out of 9 (89%) displayed DOG1-positive immunostaining. *BRAF*-mutated GIST showed a PFS of 3.45 months, while *NF1*-mutated GIST had a PFS of 0.92 months, according to central radiological assessment.

## Discussion

In this phase II trial, we found that 86.7% of 15 patients treated with regorafenib, mainly as upfront therapy of advanced disease, had DCR at 12 weeks, while 13% and 54% had partial response according to RECIST and Choi criteria respectively and according to central radiology review. Moreover, the 20% decrease in median tumor density, and the fact that 60% of patients experienced some tumor shrinkage suggest activity of regorafenib in KP-wGIST, even when the study was prematurely closed.

The activity of imatinib reported in the context of KP-wGIST has fluctuated from 23% in the pre-NGS era [8] to 2% in the post-NGS era [4]. In another study, after resequencing with NGS, authors reported 1 (8%) partial response in 12 SDH-mutant GIST patients treated with imatinib [9]. Progression-free survival reached with imatinib also seemed overemphasized in the pre-NGS period, 13 months,[2] compared with 9 months in the subset of SDH-deficient KP-wGIST in the post-NGS era [9]. Even when no clear data is available regarding DCR in KP-wGIST patients sequenced by NGS and treated with imatinib, 73% could also be overemphasized [8]. In any case, this outcome is lower than 100% for DCR, achieved with regorafenib in our study. Indeed, DCR ≥ 12 weeks seems to be an unsatisfactory endpoint in the context of certain indolent disease such as KP-wGIST. Another series with 6 SDH-deficient GIST patients also found a 100% clinical benefit rate [10]. Neither do we have prospective data of Choi responses with imatinib in KP-wGIST patients screened by NGS, which makes it more difficult to make indirect comparisons. Retrospective data on regorafenib in 28 GIST patients found 29% Choi partial responses, which was clearly superior to the 4% detected by RECIST. In that series, 2 out of 3 KP-wGIST reached partial response according to Choi criteria [11].

When we consider the 9 SDH-deficient patients in our series, 2 (22%) RECIST partial responses and 7 (78%) cases of stable disease were centrally detected. All of them were gastric and almost universally expressed positive-KIT and DOG1-positive immunostaining. In line with our findings, regorafenib achieved a median PFS of 42.9 months in a retrospective series of SDH-deficient and likely SDH-deficient GIST. In the same study, authors reported a median PFS of 14.7 and 18 months for imatinib and sunitinib respectively [12]. A phase II trial with regorafenib in advanced and progressing GIST patients, after imatinib and sunitinib failure, included 6 SDH-deficient GIST that obtained a clinical benefit rate of 100% and 2 of them reached a partial response [10]. A phase II trial testing linsitinib reported a 9-month PFS rate of 52% and a 9-month CBR of 40% in 15 SDH-deficient GIST patients. Our results with regorafenib were 67% for both outcomes, comparing favorably with linsitinib. Furthermore, it has been published that patients harboring SDH mutants involving subunit A had a longer overall survival [13]. We could not confirm this difference, since only 3 cases with this mutation were detected in our series.

Sunitinib induced no responses and provided a clinical benefit rate of 56% in 9 patients classified as KP-wGIST. The trial explored sunitinib in phase I/II with imatinib-resistant GIST patients [14]. By contrast, 4 of 38 (10.5%) patients with SDH-deficient GIST patients from a retrospective observational study experienced RECIST responses with sunitinib [4]. Pazopanib induced disease control over 17 months in a SDH-deficient GIST patient included in a phase I/II trial, another similar patient dropped out the study in the first cycle due to toxicity [15]. Together with the previous information, we could speculate that drugs with antiangiogenic properties exhibit more activity in SDH-deficient KP-wGIST than imatinib. The longer PFS, statistically significant by Choi criteria in our study, and OS, not reaching statistical significance probably due to the low number of cases, favoring the SDH-deficient subset could be due to the more indolent tumor biology and not necessarily to the antiangiogenic effect.

The fact that 47% of supposed KP-wGIST by Sanger were really *KIT* or *PDGFRα* mutant GISTs after NGS sequencing has hindered the expectancy of recruitment and, in the end, this has resulted in a premature closure of the study. Likewise, other authors found substantial discrepancies between Sanger and NGS sequencing for classifying KP-wGIST. In fact, Sanger sequencing overestimates KP-wGIST in a range of between 20% and 41% [16]. Actually, some cases of good and long-responders to imatinib in the context of supposedly KP-wGIST were, in reality, *KIT* (or *PDGFRA*) mutant GISTs after deeper sequencing. Moreover, some GIST complex insertions/deletions, among other genomic alterations, could not be identified by routine NGS.

Another limitation of the study was the low compliance regarding quality-of-life questionnaires, probably related to the academic nature of the study, due to budget constraints limiting on-site monitoring. The almost universal temporal discontinuations (93%) indicated that the treatment is toxic and required reduction adjustments in almost half of the patients (53%). This was in line with the accumulated experience with regorafenib in the third line of advanced GIST patients, where it is difficult to maintain the dose of 160 mg per day.

In conclusion, KP-wGIST represents an uncommon subset within GIST patients, rarer than initially expected using Sanger sequencing. Within this heterogeneous population, regorafenib activity compares favorably with other tyrosine kinase inhibitors especially in the SDH-deficient GIST subset, and consequently it should be taken into consideration as upfront therapy of advanced KP-wGIST patients.

### Electronic supplementary material

Below is the link to the electronic supplementary material.

Supplementary Material 1

Supplementary Material 2

Supplementary Material 3

Supplementary Material 4

Supplementary Material 5

Supplementary Material 6

Supplementary Material 7

Supplementary Material 8

Supplementary Material 9

## Data Availability

Not applicable.

## List of abbreviations

DCR: Disease control rate
ECOG: Eastern Cooperative Oncology Group
FH: Fumarate hydratase
GIST: Gastrointestinal stromal tumors
HU: Hounsfield units
ICC: Interstitial Cajal cell
KP-wGIST: *KIT /PDGFRA* wild-type GIST
NGS: Next generation sequencing
ORR: Overall response rate
OS: Overall survival
PFS: Progression-free survival
SDH: Succinate dehydrogenase
VEGFR: Vascular endothelial growth factors
