# Supplementary figures and images for: REGISTRI: Regorafenib in first-line of KIT/PDGFRA wild type metastatic GIST: a collaborative Spanish (GEIS), Italian (ISG) and French Sarcoma Group (FSG) phase II trial
Source: Mol Cancer. 2023 Aug 9;22:127. doi: 10.1186/s12943-023-01832-9 (PMC10413507; doi:10.1186/s12943-023-01832-9)

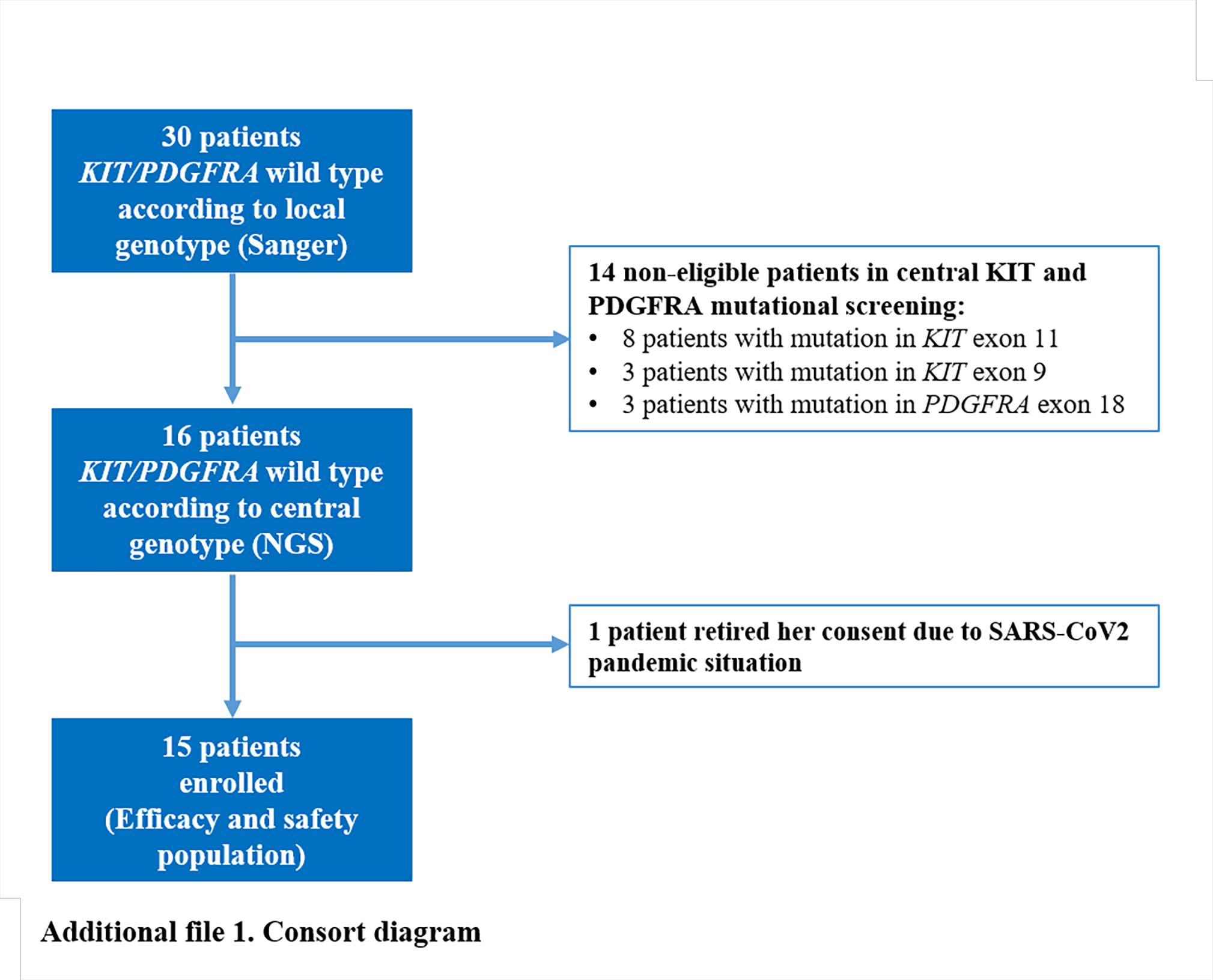

Supplement: Supplementary file 1 — Supplementary Material 1 [file 12943_2023_1832_MOESM1_ESM.tif]

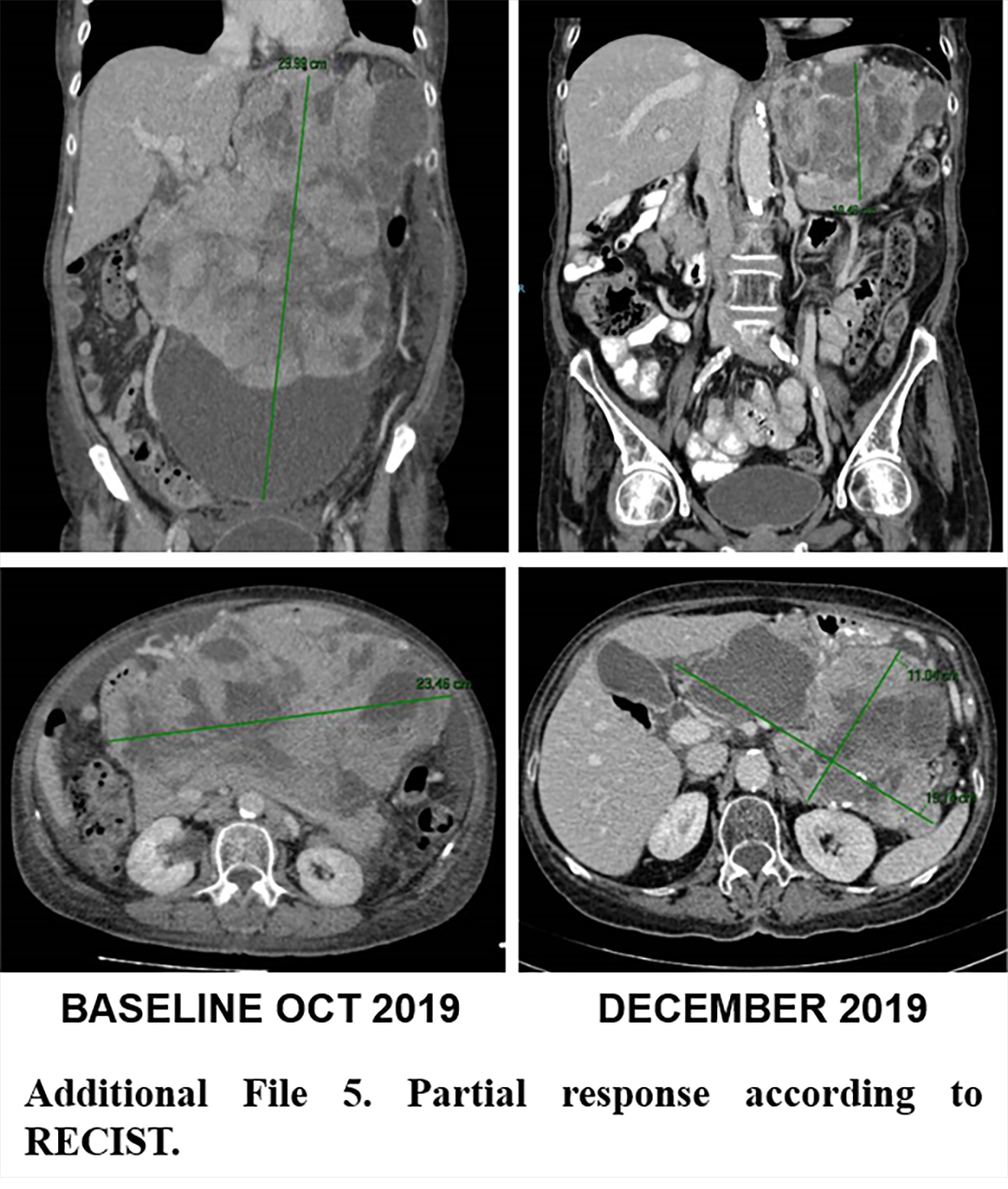

Supplement: Supplementary file 5 — Supplementary Material 5 [file 12943_2023_1832_MOESM5_ESM.tif]

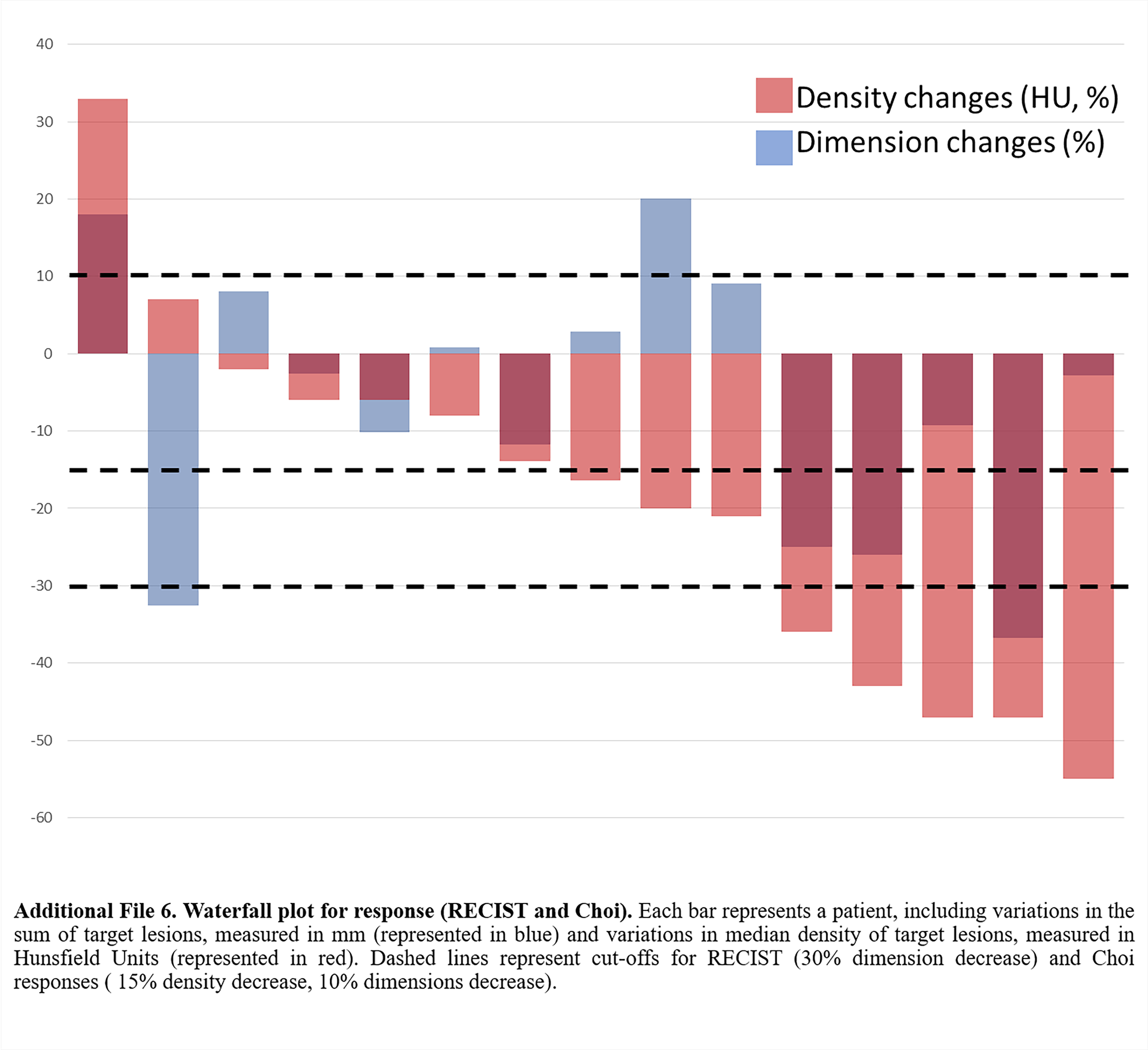

Supplement: Supplementary file 6 — Supplementary Material 6 [file 12943_2023_1832_MOESM6_ESM.tif]

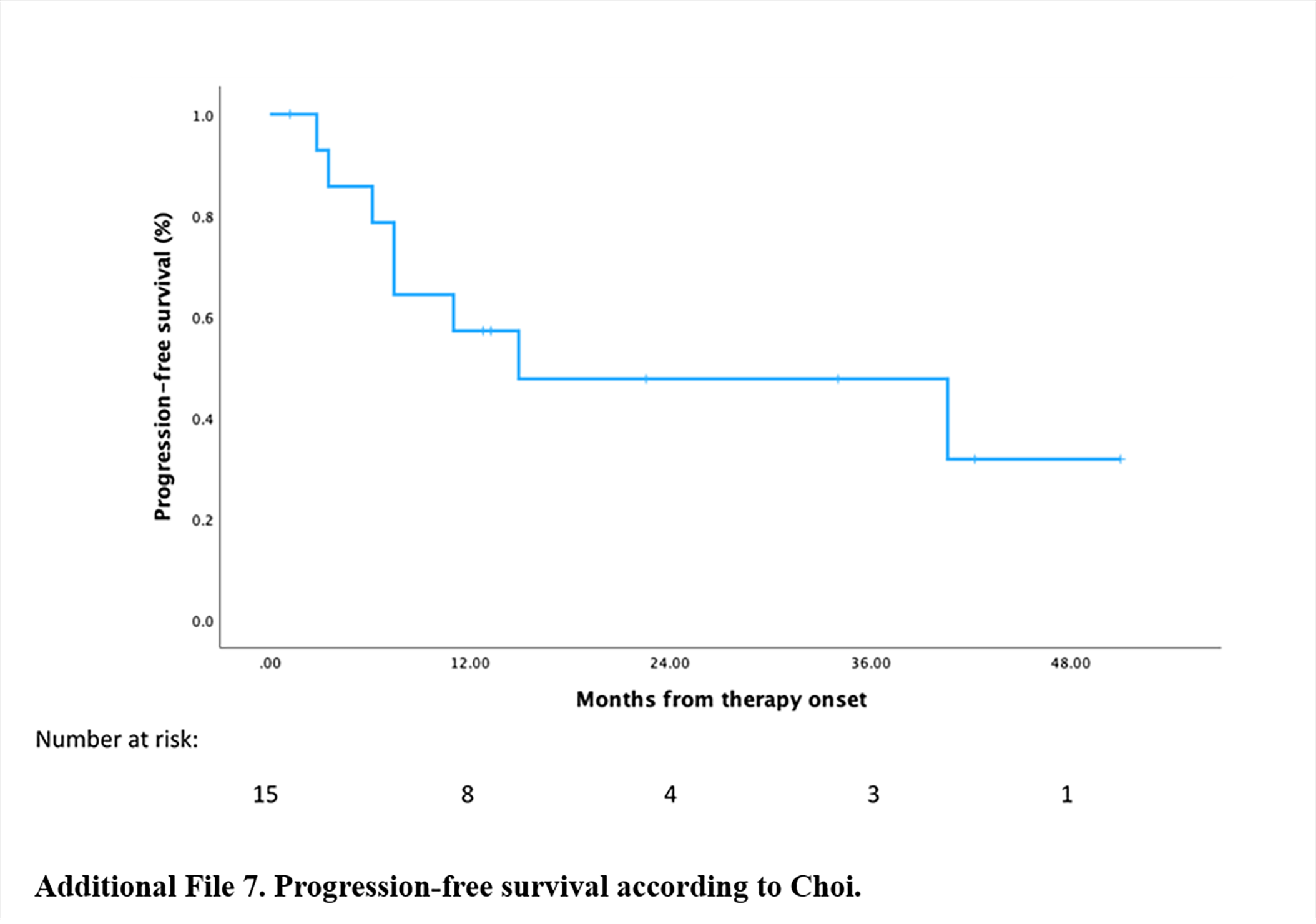

Supplement: Supplementary file 7 — Supplementary Material 7 [file 12943_2023_1832_MOESM7_ESM.tif]

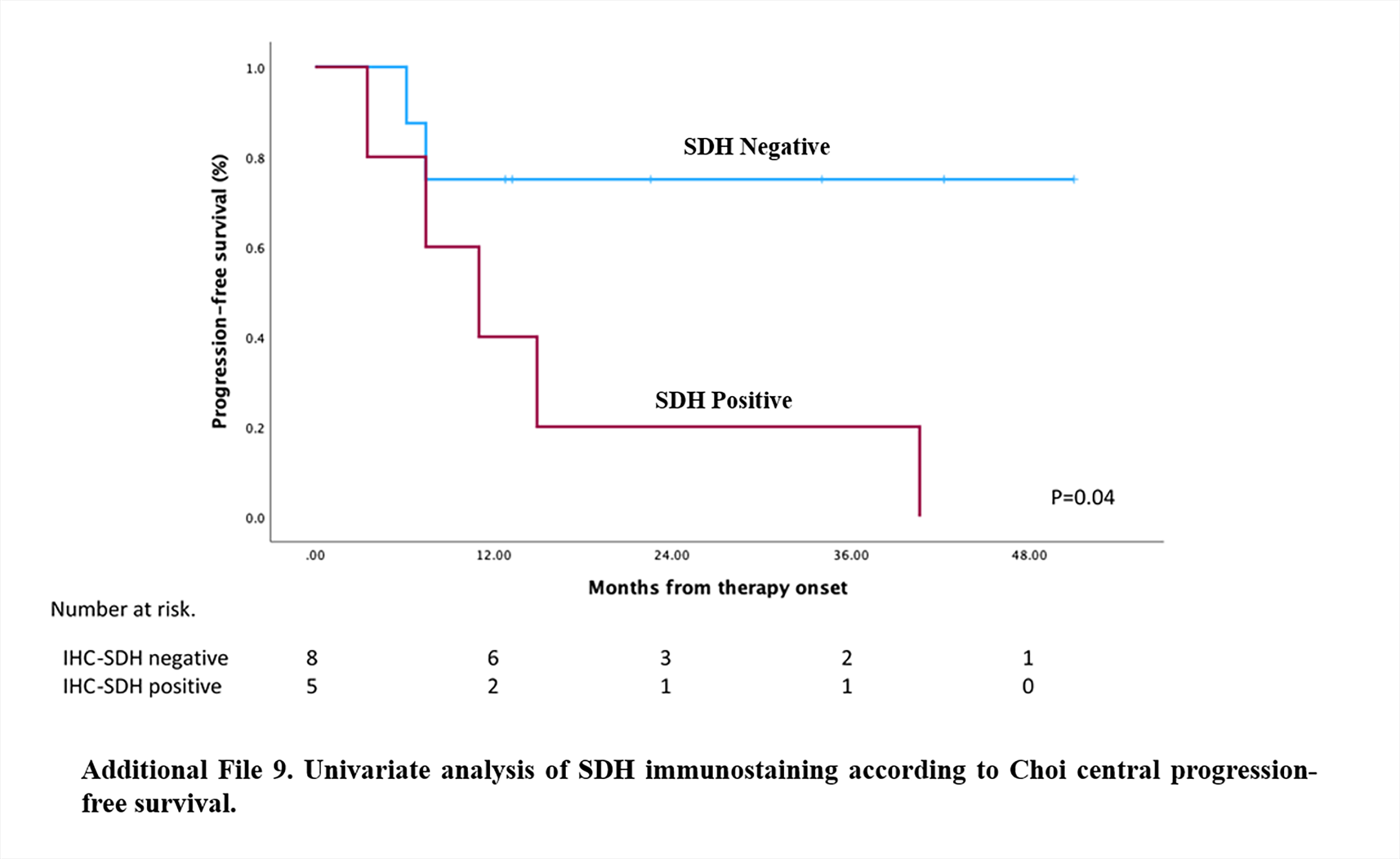

Supplement: Supplementary file 9 — Supplementary Material 9 [file 12943_2023_1832_MOESM9_ESM.tif]
