# Supplementary material for: REGISTRI: Regorafenib in first-line of KIT/PDGFRA wild type metastatic GIST: a collaborative Spanish (GEIS), Italian (ISG) and French Sarcoma Group (FSG) phase II trial
Source: Mol Cancer. 2023 Aug 9;22:127. doi: 10.1186/s12943-023-01832-9 (PMC10413507; doi:10.1186/s12943-023-01832-9)
Supplement: Supplementary file 2 — Supplementary Material 2 [file 12943_2023_1832_MOESM2_ESM.docx]

Additional File 2 – SDH immunohistochemistry and mutational screening

| ID | SDHB immunostaining | Mutational screening | Any alteration in SHD |
| --- | --- | --- | --- |
| 01 | Positive | *MAX* (c.295+1G>A) | No |
| 02 | Not evaluable | *NF1* (c.3870+14A>G) | No |
| 03 | Not evaluable | *SDHB* (c.744C>G, p.Asn248Lys) | Yes |
| 04 | Negative | *SDHA* (C923T p.T308M + c.1799G>A R600Q exon 8+14) | Yes |
| 05 | Positive | Not applicable | No |
| 06 | Positive | Not applicable | No |
| 07 | Negative | *FH* (c.707C>T, p.Thr236Ile, rs773382103) – VUS (Variant of Uncertain Significance according to ClinVar) | Yes |
| 08 | Negative | *SDHC* (c.1A>G) | Yes |
| 09 | Negative | Not applicable | Yes |
| 10 | Negative | *SDHC* (c.379C>T, p.His127Tyr) and *SDHD* (VUS c.22A>G, p.Ser 8Gly) | Yes |
| 11 | Positive | Not evaluable | No |
| 12 | Negative | *SDHA* (c.1103T>G, p.Leu368Arg, c.428C>T, p.Thr143Met) | Yes |
| 13 | Negative | Not applicable | Yes |
| 14 | Positive | *BRAF* (c.1799T>A, p.Val600Glu) | No |
| 15 | Negative | *SDHA* (c.1334C>T, p.Ser445Leu) | Yes |
