# Supplementary material for: REGISTRI: Regorafenib in first-line of KIT/PDGFRA wild type metastatic GIST: a collaborative Spanish (GEIS), Italian (ISG) and French Sarcoma Group (FSG) phase II trial
Source: Mol Cancer. 2023 Aug 9;22:127. doi: 10.1186/s12943-023-01832-9 (PMC10413507; doi:10.1186/s12943-023-01832-9)
Supplement: Supplementary file 3 — Supplementary Material 3 [file 12943_2023_1832_MOESM3_ESM.docx]

Additional File 3 – Safety profile (n=15)

| EVENT | Any Grade | Grade 1-2 | Grade 3 | Grade 4 |
| --- | --- | --- | --- | --- |
| Hematological | | | | |
| Anemia | 3 (20.0%) | 1 (6.7%) | 2 (13.3%) | 0 |
| Lymphocytopenia | 2 (13.3%) | 2 (13.3%) | 0 | 0 |
| Neutropenia | 1 (6.7%) | 1 (6.7%) | 0 | 0 |
| Thrombocytopenia | 1 (6.7%) | 1 (6.7%) | 0 | 0 |
| Non-Hematological | | | | |
| Hypertension | 10 (66.7%) | 8 (53.3%) | 2 (13.3%) | 0 |
| Fatigue | 9 (60.0%) | 9 (60.0%) | 0 | 0 |
| Hand-foot syndrome | 8 (53.3%) | 6 (40.0%) | 2 (13.3%) | 0 |
| Diarrhea | 7 (46.7%) | 6 (40.0%) | 1 (6.7%) | 0 |
| Anorexia | 6 (40.0%) | 4 (26.7%) | 2 (13.3%) | 0 |
| Rash | 6 (40.0%) | 5 (33.3%) | 1 (6.7%) | 0 |
| Mucositis oral | 5 (33.3%) | 5 (33.3%) | 0 | 0 |
| ALT/SGPT increased | 4 (26.7%) | 1 (6.7%) | 3 (20.0%) | 0 |
| Alopecia | 4 (26.7%) | 4 (26.7%) | 0 | 0 |
| Constipation | 4 (26.7%) | 4 (26.7%) | 0 | 0 |
| Skin Disorder | 4 (26.7%) | 3 (20.0%) | 0 | 1 (6.7%) |
| AST/SGOT increased | 3 (20.0%) | 1 (6.7%) | 2 (13.3%) | 0 |
| Dysgeusia | 3 (20.0%) | 3 (20.0%) | 0 | 0 |
| GGT increased | 3 (20.0%) | 2 (13.3%) | 1 (6.7%) | 0 |
| Headache | 3 (20.0%) | 3 (20.0%) | 0 | 0 |
| Pruritus | 3 (20.0%) | 2 (13.3%) | 0 | 1 (6.7%) |
| ALP increased | 2 (13.3%) | 1 (6.7%) | 1 (6.7%) | 0 |
| Abdominal pain | 2 (13.3%) | 2 (13.3%) | 0 | 0 |
| Blood bilirubin increased | 2 (13.3%) | 1 (6.7%) | 1 (6.7%) | 0 |
| Vomiting | 2 (13.3%) | 2 (13.3%) | 0 | 0 |
| Aphonia | 1 (6.7%) | 1 (6.7%) | 0 | 0 |
| Arthralgia | 1 (6.7%) | 1 (6.7%) | 0 | 0 |
| Conjunctivitis | 1 (6.7%) | 1 (6.7%) | 0 | 0 |
| Cough | 1 (6.7%) | 1 (6.7%) | 0 | 0 |
| Dizziness | 1 (6.7%) | 1 (6.7%) | 0 | 0 |
| Dysesthesia | 1 (6.7%) | 1 (6.7%) | 0 | 0 |
| Dyspepsia | 1 (6.7%) | 1 (6.7%) | 0 | 0 |
| Fever | 1 (6.7%) | 1 (6.7%) | 0 | 0 |
| Flushing | 1 (6.7%) | 1 (6.7%) | 0 | 0 |
| Hemorrhoids | 1 (6.7%) | 1 (6.7%) | 0 | 0 |
| Hypertransaminasemia | 1 (6.7%) | 1 (6.7%) | 0 | 0 |
| Hypocalcemia | 1 (6.7%) | 1 (6.7%) | 0 | 0 |
| Hypoglycemia | 1 (6.7%) | 1 (6.7%) | 0 | 0 |
| Hypokalemia | 1 (6.7%) | 0 | 1 (6.7%) | 0 |
| Hypothyroidism | 1 (6.7%) | 1 (6.7%) | 0 | 0 |
| Inferior members edema | 1 (6.7%) | 1 (6.7%) | 0 | 0 |
| Mucosa dryness | 1 (6.7%) | 1 (6.7%) | 0 | 0 |
| Myalgia | 1 (6.7%) | 1 (6.7%) | 0 | 0 |
| Nausea | 1 (6.7%) | 1 (6.7%) | 0 | 0 |
| Serum amylase increased | 1 (6.7%) | 1 (6.7%) | 0 | 0 |
