# Supplementary material for: REGISTRI: Regorafenib in first-line of KIT/PDGFRA wild type metastatic GIST: a collaborative Spanish (GEIS), Italian (ISG) and French Sarcoma Group (FSG) phase II trial
Source: Mol Cancer. 2023 Aug 9;22:127. doi: 10.1186/s12943-023-01832-9 (PMC10413507; doi:10.1186/s12943-023-01832-9)
Supplement: Supplementary file 4 — Supplementary Material 4 [file 12943_2023_1832_MOESM4_ESM.docx]

Additional File 4 - Quality of life analysis

| **Functional Scales** | Median baseline (range) | Median 2^nd^ (range) | Difference (range) | p |
| --- | --- | --- | --- | --- |
| Physical functioning | 93 (46 to 100) | 100 (60 to 100) | 3.5 (-26 to 14) | 0.5 |
| Role functioning | 83 (0 to 100) | 100 (33 to 100) | 0 (-17 to 50) | 0.14 |
| Emotional functioning | 87 (75 to 100) | 83 (66 to 100) | 0 (-17 to 0) | 0.1 |
| Cognitive functioning | 100 (83 to 100) | 91 (67 to 100) | 0 (-33 to 17) | 0.48 |
| Social functioning | 74 (33 to 100) | 91 (33 to 100) | 0 (-17 to 34) | 0.26 |
| **Symptom Scale** | Median baseline (range) | Median 2^nd^ (range) | Difference (range) | p |
| Fatigue | 20 (0 to 56) | 21 (0 to 67) | 0 (-20 to 47) | 0.42 |
| Nausea & Vomiting | 0 (0 to 17) | 0 (0 to 17) | 0 (-17 to 0) | 0.32 |
| Pain | 25 (0 to 100) | 17 (0 to 67) | -8 (-33 to 47) | 0.8 |
| Dyspnea | 0 (0 to 33) | 0 (0 to 66) | 0 (0 to 33) | 0.16 |
| Insomnia | 33 (0 to 33) | 16 (0 to 33) | 0 (-33 to 33) | 0.56 |
| Appetite loss | 33 (0 to 33) | 0 (0 to 33) | 0 (-33 to 0) | 0.16 |
| Constipation | 0 (0 to 33) | 0 (0 to 66) | 0 (0 to 33) | 0.32 |
| Diarrhea | 0 (0 to 0) | 0 (0 to 33) | 0 (0 to 33) | 0.16 |
| Financial difficulties | 16 (0 to 66) | 0 (0 to 66) | 0 (-66 to 0) | 0.32 |
| **Global Health Status** | 67 (50 to 100) | 79 (25 to 83) | 0 (-25 to 17) | 0.67 |
