# Supplementary material for: REGISTRI: Regorafenib in first-line of KIT/PDGFRA wild type metastatic GIST: a collaborative Spanish (GEIS), Italian (ISG) and French Sarcoma Group (FSG) phase II trial
Source: Mol Cancer. 2023 Aug 9;22:127. doi: 10.1186/s12943-023-01832-9 (PMC10413507; doi:10.1186/s12943-023-01832-9)
Supplement: Supplementary file 8 — Supplementary Material 8 [file 12943_2023_1832_MOESM8_ESM.docx]

Additional File 8. Univariate analysis

|  | Median PFS  (95% CI)  (Local) | p | Median PFS  (95% CI)  (Central CHOI) | p | Median PFS  (95% CI)  (Central RECIST) | p | Median OS  (95% CI) | p |
| --- | --- | --- | --- | --- | --- | --- | --- | --- |
| Median age at enrollment:   - 16-57 - >57 | 21.1 (0-58.2)  18.4 (5-31.9) | 0.25 | 7.4 (0-38.3)  14.9 (9.1-20.7) | 0.88 | 8.9 (0-21.1)  11 (5-16.9) | 0.9 | NR  28.7 (11.4-46) | 0.041 |
| Gender:   - Male - Female | 33.4 (2.7-64.1)  14.4 (0-31.4) | 0.11 | 7.4 (1.6-13.3)  NR | 0.2 | 8.7 (4.4-13)  14.9 (NA) | 0.31 | NR  34 (25.9-42.1) | 0.92 |
| ECOG at Baseline:   - 0 - 1 | 20.6 (16.8-24.3)  6.1 (0-13.1) | 0.25 | 14.9 (0-41.3)  6.1 (NA) | 0.9 | 14.9 (7.9-21.9)  4.4 (0-10.2) | 0.29 | 34 (NA)  12.8 (0-33.2) | 0.23 |
| Median tumor size at diagnosis:   - 0-60 - >60 | 21 (0-43.4)  18.4 (0-48.6) | 0.31 | 40.6 (0-105)  11 (3-18.2) | 0.4 | 8.9 (0-19.3)  11 (3.8-18.2) | 0.76 | NR  28.7 (4.8-52.6) | 0.054 |
| Tumor extension at diagnosis:   - Localized - Locally advanced - Metastatic | 9 (0-32.1)  NR  10.1 (1.2-40.9) | 0.78 | 11 (NA)  40.6 (NA)  7.4 (0-15.7) | 0.79 | 11  4.4 (NA)  8.9 (1.7-16) | 0.63 | NR  12.8 (NA)  NR | 0.81 |
| Tumor extension at enrollment:   - Locally advanced - Metastatic | 6.1 (NA)  20.6 (10.6-30.5) | 0.48 | 40.5 (NA)  11 (0-22.5) | 0.61 | 4.4 (NA)  11 (1.7-20.3) | 0.47 | 12.8 (NA)  34 (NA) | 0.86 |
| TRANSLATIONAL RESEARCH | | | | | | | | |
| IHC-SDH:   - Positive - Negative | 20.6 (16-25.1)  30.5 (0-70.1) | 0.63 | 11 (3.3-18.6)  NR | 0.04 | 11 (6.4-15.6)  8.9 (NA) | 0.11 | 28.7 (NA)  NR | 0.49 |
| Any mutation:   - Yes - No | 14.4 (0-31)  20.6 (0-49.6) | 0.52 | NR  14.9 (0-37.4) | 0.67 | 11 (0.6-21.4)  14.9 (7.5-22.3) | 0.93 | 34 (6.5-61.5)  NR | 0.31 |
| SHD mutation:   - SDH - Other gene - No mutation | 8.4 (0-30.8)  1.7 (0-19.2)  20.6 (0-49.6) | 0.26 | NR  11 (0-23)  14.9 (0-37.4) | 0.77 | 7.4 (NA)  11 (0-23)  14.9 (7.5-22.3) | 0.97 | 34 (2.2-65.9)  20.3 (0-48.8)  NR | 0.57 |
| Any alteration in any gene:   - Yes - No | 14.4 (0-32.9)  21.1 (20.3-21.9) | 0.43 | NR  14.9 (2.9-26.8) | 0.48 | 11  14.9 (5.2-24.5) | 0.64 | NR  34 (NA) | 0.62 |
| Any alteration in SDH genes:   - Yes - No | 30.5 (0-61.8)  18.4 (0-41.1) | 0.53 | NR  11 (3.3-18.7) | 0.099 | NR  11 (6.4-15.6) | 0.20 | NR  28.7 (11.1-46.3) | 0.45 |
| Type of SDH alteration (n=11):   - Mutation - Other | 14.4 (0-30.8)  20.6 (16-25.1) | 0.78 | NR  11 (3.3-18.6) | 0.18 | 7.4 (NA)  11 (6.4-15.6) | 0.39 | 34 (23.7-44.4)  28.7 (NA) | 0.74 |
| KIT_IHC:   - Positive - Negative | 14.4 (0-32.9)  21.1 (20.3-21.9) | 0.43 | NR  14.9 (2.9-26.8) | 0.48 | 11 (NA)  14.9 (5.2-24.5) | 0.64 | 34 (NA)  NR | 0.62 |
| DOG1_IHC:   - Positive - Negative | 21.1 (0-45.9)  20.6 (NA) | 0.61 | 40.6 (0-101.4)  14.9 (NA) | 0.5 | 16.2 (4.8-27.6)  14.9 (NA) | 0.68 | NR  28.7 (NA) | 0.16 |
